# A Novel Isothermal Compression Method for Energy Conservation in Fluid Power Systems

**DOI:** 10.3390/e22091015

**Published:** 2020-09-11

**Authors:** Teng Ren, Weiqing Xu, Guan-Wei Jia, Maolin Cai

**Affiliations:** 1 School of Automation Science and Electrical Engineering, Beihang University, Beijing 100191, China; ren_teng@buaa.edu.cn (T.R.); caimaolin@buaa.edu.cn (M.C.); 2 Pneumatic and Thermodynamic Energy Storage and Supply Beijing Key Laboratory, Beijing 100191, China; 3 School of Physics and Electronics, Henan University, Kaifeng 475004, China; jiaguanwei@buaa.edu.cn

**Keywords:** isothermal piston, isothermal compression, energy conservation, fluid power systems

## Abstract

Reducing carbon emissions is an urgent problem around the world while facing the energy and environmental crises. Whatever progress has been made in renewable energy research, efforts made to energy-saving technology is always necessary. The energy consumption from fluid power systems of industrial processes is considerable, especially for pneumatic systems. A novel isothermal compression method was proposed to lower the energy consumption of compressors. A porous medium was introduced to compose an isothermal piston. The porous medium was located beneath a conventional piston, and gradually immerged into the liquid during compression. The compression heat was absorbed by the porous medium, and finally conducted with the liquid at the chamber bottom. The heat transfer can be significantly enhanced due to the large surface area of the porous medium. As the liquid has a large heat capacity, the liquid temperature can maintain constant through circulation outside. This create near-isothermal compression, which minimizes energy loss in the form of heat, which cannot be recovered. There will be mass loss of the air due to dissolution and leakage. Therefore, the dissolution and leakage amount of gas are compensated for in this method. Gas is dissolved into liquid with the pressure increasing, which leads to mass loss of the gas. With a pressure ratio of 4:1 and a rotational speed of 100 rpm, the isothermal piston decreased the energy consumption by 45% over the conventional reciprocation piston. This gain was accomplished by increasing the heat transfer during the gas compression by increasing the surface area to volume ratio in the compression chamber. Frictional forces between the porous medium and liquid was presented. Work to overcome the frictional forces is negligible (0.21% of the total compression work) under the current operating condition.

## 1. Introduction

With the acceleration of industrialization, man’s demand for energy has increased, and serious environmental pollution and ecological destruction problems have been brought along with it such as global environmental problems caused by coal and petroleum-based fossil energy including acid rain, ozone layer destruction, haze weather, and so on. These are the consequences of increased carbon emissions from the burning of fossil fuels. For this reason, reducing carbon emissions is an urgent problem to be solved worldwide. In 2019, global greenhouse gas emissions were still as high as 33 billion tons [1].

Three methods can be used to reduce carbon emissions. The first is to continuously develop energy-saving technologies [2]. Currently, industrial energy consumption is relatively high, energy efficiency is relatively low, and there is still much room for energy saving potentials [3]. The second is to develop clean new energy technologies to eliminate carbon emissions from the source. The third is carbon capture and storage (CCS), which is one of the new environmental technologies aiming to decrease the emission of CO_2_ [4].

Several energy sources that can possibly replace fossil fuels such as bioenergy [5], hydropower [6], and nuclear power [7] also face many problems and challenges in production and consumption [8]. In the production process of bioenergy, its environmental cost is much higher than fossil energy [9]. The construction of hydropower stations will destroy the natural attributes of the river [10]. Nuclear energy is highly efficient and clean [11], however, the issue of nuclear leakage, the nuclear pollution caused by nuclear energy and the proper disposal of nuclear waste are very difficult issues to resolve, which have also severely restricted the development of nuclear energy [12]. Other renewable energy sources such as solar energy, wind energy, geothermal energy, and tidal energy, are unevenly distributed in time and space and are intermittent [13]. In China, these alternative energy sources are only a useful supplement to the fossil energy system and cannot replace existing fossil energy on a large scale. At present, fossil energy will continue to be the basis for human survival and development for a long time to come.

For CCS, although it has been technically feasible for more than ten years [14], and the importance of commercial deployment of CCS has been widely acknowledged by many countries [15], it is still lagging in the demonstration stage at a small-scale thus far [16]. Therefore, to reduce carbon emissions, energy saving technologies are extremely important.

**Figure 1:**
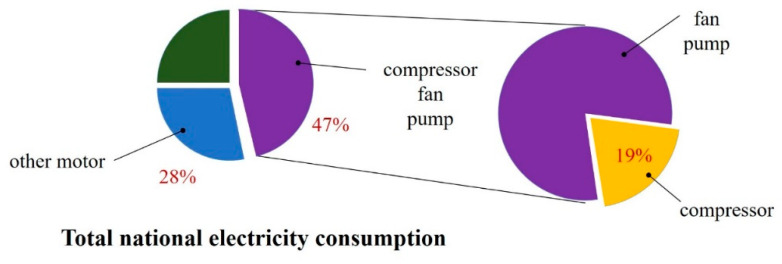
Electricity consumption statistics of compressors in China.

The amount of carbon emissions from the fluid power systems of industrial processes is considerable, especially for pneumatic systems [17]. In China, the electricity consumption for fluid machineries (compressors, fans, and pumps) is much larger (47%) than that for other motors (28%). About 19% of the energy consumption of fluid machineries is for compressors. Therefore, compressors account for about 9% of the total national electricity consumption, as shown in Figure 1 [18]. From this point of view, energy saving technology is recognized as the key technology for energy savings of a pneumatic system [19].

**Figure 2:**
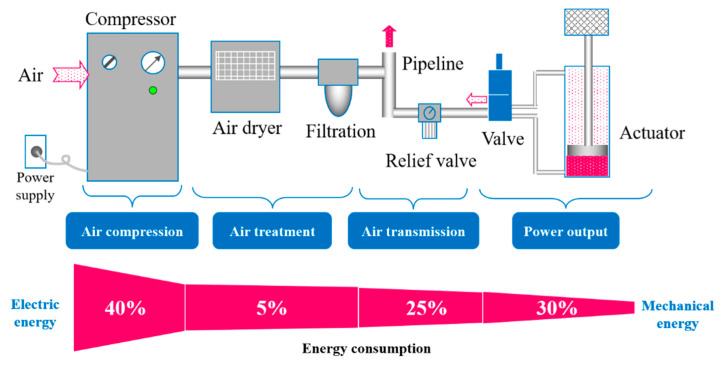
Composition and energy distribution of a pneumatic system.

Pneumatic systems consist of air compression, air treatment, air transmission, and execution. The distribution of energy consumption in each link of the pneumatic system is shown in Figure 2. The air compression process accounts for 40% of the total energy consumption, where the air treatment process accounts for 5% and air transmission accounts for 25%. The execution process only accounts for 30% of the total energy. The air compression accounts for most of the total energy consumption.

**Figure 3:**
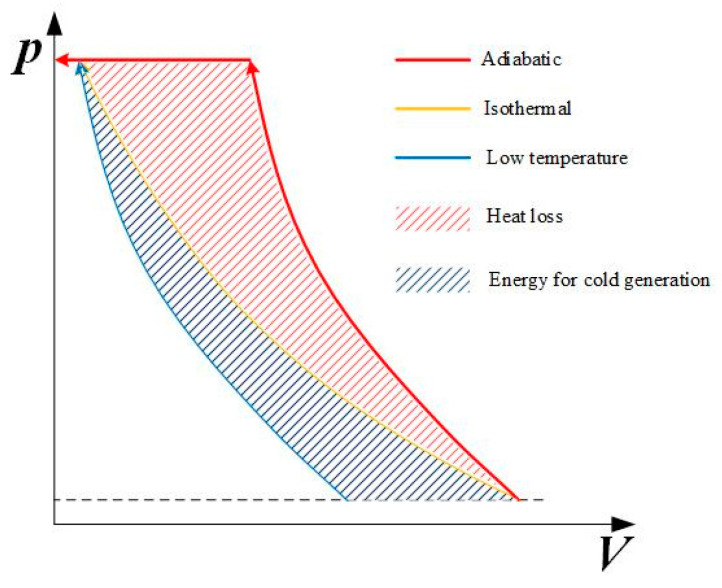
*p–V* diagram for gas compression.

Air is compressed in the compressor. To save energy, air can be compressed by way of adiabatic, isothermal, and low temperature. As shown in Figure 3, most compressors work under near adiabatic conditions, and the compression work consumption is the most. Though it could be stored as a form of heat, the cycle efficiency of compressors is limited by the poor heat transfer. To increase the compression efficiency, the heat of compression should be prevented, therefore, isothermal compression and low-temperature compression.

The isothermal approach is used to enhance the heat transfer of compressed air to maintain the temperature constant as the environment. Two main methods to achieve isothermal compression are to inject the liquid spray or foam into the compression chamber and liquid piston. For liquid spray injection, it can increase the heat transfer area because of the micro droplet area. This method was first proposed by Coney [20]. By injecting the water spray into the compression chamber of a reciprocating compressor, the compression work can be reduced by 28% [21]. Foam can also be injected into the compression chamber with a mixture of air and water [22]. Injecting foam instead of water spray can lower the required energy for spray generation and can stay longer in the compression chamber. However, additional energy consumption is required to generate higher pressure (3~5 MPa) for micro droplets. Energy consumption of the micro spray droplets is equal to the work conservation when the water spray mass reaches 1.5 g/cycle (nozzle in diameter of 0.3 mm) [23].

For a liquid piston, gas is compressed directly by a column of liquid in a chamber of fixed volume. It can be found that heat transfer is improved by using this proposed liquid piston compared with the conventional reciprocating piston through experimental investigation [24,25,26]. The heat transfer area can be improved for adaption to a gas chamber with an irregular shape. Air leakage and friction resistance can also be lowered [27]. However, the weakness is that air will be mixed with the liquid as the liquid piston is driven by the hydraulic system. Other problems such as noise, vibration, and intensive wear will also arise.

Low-temperature compression refers to lowering the air temperature at the inlet of the compressor by low-temperature refrigeration devices, which can reduce compression heat and energy consumption. Extra energy consumption is needed to make the cold source, which is the area enclosed by the blue line and yellow line in Figure 3. Furthermore, low temperatures can cause frost, which can harm refrigeration devices.

This paper proposes a novel concept to achieve isothermal compression: isothermal piston. This can be applied in industrial compressors and refrigeration to increase the energy efficiency. Therefore, the energy consumption in fluid systems can be lowered significantly, and the dead volume can be compensated due to the mobility of the fluid.

## 2. Concept of the Isothermal Piston

**Figure 4:**
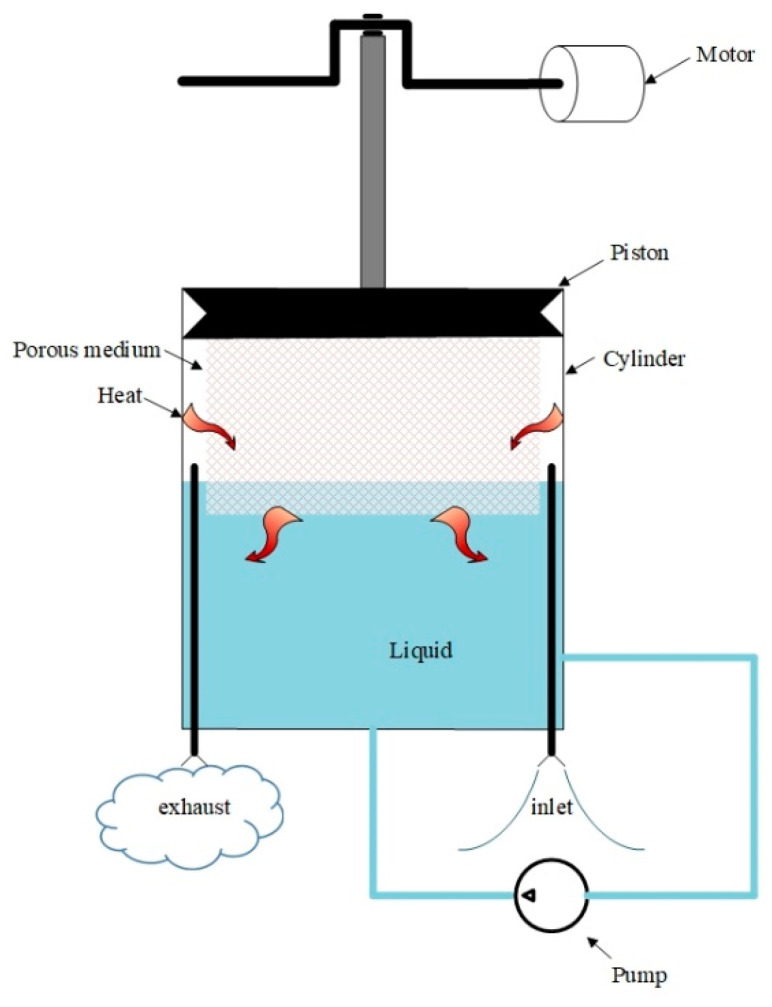
Principle of isothermal piston.

The isothermal piston is composed of a conventional piston and porous medium [28], as shown in Figure 4. A certain amount of liquid is in the compression chamber, forming a gas–solid–liquid coupled structure to enhance heat transfer. When compressed, the compression heat of compressed air is transferred to the liquid through the porous medium. The liquid temperature is almost constant due to the large heat capacity. The pump can be applied in practical application to circulate the water to dissipate heat to the environment through an external heat exchanger.

**Figure 5:**
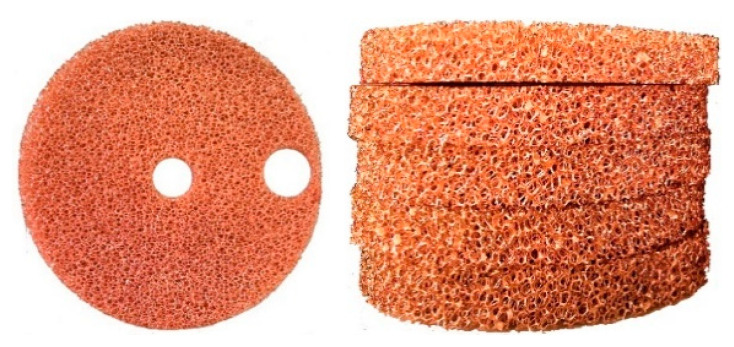
Porous medium.

The porous medium has a large specific surface area because of its special structure, which can enhance the heat transfer, as shown in Figure 5. The porous medium in this study was copper foam, which consists of pores inside the copper that are foamy. As copper has good conductivity, it can absorb a large amount of compression heat. The property of the copper foam is presented in Table 1.

## 3. Mathematical Model and Experimental Setup

Based on the conventional piston and combined with the porous heat transfer medium, the concept of an isothermal piston is proposed. Compression by an isothermal piston can enhance heat exchange, greatly reduce the air temperature in the compression chamber, and approach quasi-isothermal compression. At the same time, the introduction of porous media adds flow resistance to the piston and increases the complexity of the flow process.

The internal energy of the air is determined by the work done by the piston (*δW*), the heat transferred to the system, and the enthalpy (*dH_dis_*) transferred to the water. As there will be mass loss (discussed later), enthalpy should be introduced to describe the energy of air that dissolves into the water with compression. The compression process of air can be modeled by the state equation and energy equation as follows:

(1) $$\left\{ \begin{array}{l} p\cdot V=m\cdot R\cdot T\\ dU=dH_{dis}+\delta Q+\delta W \end{array}\right.$$

For the variation of the resistance pressure gradient caused by the relative displacement between the porous medium and the fluid, it can be determined by the Ergun surface cube model [29,30]. The change of resistance pressure gradient is mainly related to the structure of the porous medium, and is affected by the shape of pores. The pressure gradient $\frac{dp_{liq}}{dL}$ is shown by the following formula:

(2) $$\frac{dp_{liq}}{dL}=\frac{\mu }{K}\cdot u+\rho \cdot C_{c}\cdot u^{2}$$

where *dL* is on the opposite direction of the piston motion; *μ* is the dynamic viscosity; *ρ* is the density of fluids; *u* is the relative velocity between a fluid and a porous medium; *K* is the permeability of porous medium; and *C_c_* is the inertia coefficient of porous medium (or shape factor). *K* and *C* [29,30] are defined as:

(3) $$K=\frac{\varepsilon^{3}d^{2}}{a{\left(1-\varepsilon \right)}^{2}}$$

(4) $$C_{c}=b\frac{\left(1-\varepsilon \right)}{\varepsilon^{3}d}$$

where *a* is a constant related to viscosity; *b* is a constant related to inertia; *ε* is the porosity; and *d* is the hydraulic radius of a porous medium.

Substituting Equations (3) and (4) into Equation (2), the classical Ergun equation [29,30] is obtained as:

(5) $$\frac{dp_{liq}}{dL}=a\frac{{\left(1-\varepsilon \right)}^{2}\mu }{\varepsilon^{3}d^{2}}\cdot u\;+\;b\frac{\left(1-\varepsilon \right)}{\varepsilon^{3}d}\cdot \rho \cdot u^{2}$$

Air resistance is negligible as viscosity and density of air (17.9 × 10^−6^ Pa∙s, 1.18 kg/m^3^) is much smaller than that of water (1.01 × 10^−3^ Pa∙s, 1 × 10^3^ kg/m^3^). The fluid resistance discussed in this paper was all about liquid resistance. Work done by the liquid resistance is:

(6) $$W=\int_{V_{0}}^{V}\frac{dp_{liq}}{dL}\cdot l\cdot dV$$

where *l* is the relative displacement of the porous medium and liquid. With the air pressure increasing as compression proceeds, there will be loss of air mass, which dissolves into the water. Henry’s law [31] revealed the relation between the dissolved amount of air and the air pressure:

(7) $$c=H^{cp}\cdot p$$

where *c* (mol/L) is the molarity of gas dissolved into the liquid; *p* is the gas pressure above the liquid at equilibrium conditions; *H^cp^* (mol/L∙atm) is Henry’s constant and is independent of temperature.

**Figure 6:**
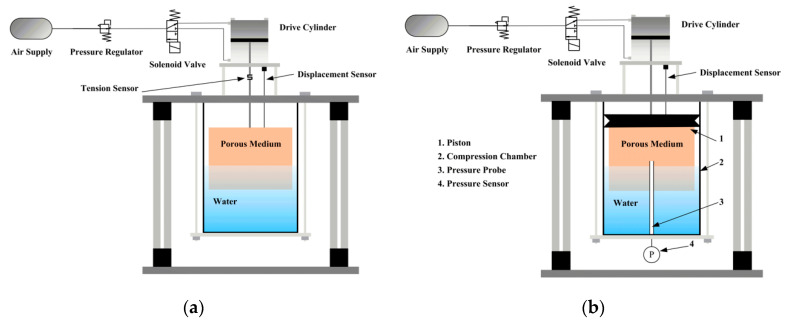
The scheme of the compression system. (**a**) Resistance measurement (**b**) configuration of the isothermal piston.

In order to measure the resistance between the porous medium and water during the operation of the isothermal piston, a tension pressure sensor is installed in the middle of the piston rod, and the resistance is obtained by the change of the data of the tension pressure sensor. The resistance measurement is shown in Figure 6a.

An air cylinder is used to drive the isothermal piston. The supply pressure of the air source was set at 0.8 MPa, and a pressure regulator connected behind was used to stabilize the force applied to the isothermal piston. The moving direction of the piston was controlled by the solenoid valve. The system was operated for one cycle in this study, and the analysis is focused on the compression process. The specific experimental scheme is depicted in Figure 6b [32].

The system has good sealing performance in a static state. There will inevitably be leakage when the piston is moving. Compensation should be analyzed in this study. Experiments without a porous medium should be conducted compared with the simulation. As the system operates only one cycle, the compression process is nearly adiabatically. The pressure difference between the experiment and simulation was caused by air leakage. According to the state equation, the amount of gas leakage can be determined when the piston is in a different position. The leakage amount at each position of the piston can be estimated by Equation (8).

(8) Δp⋅V=Δm⋅R⋅T

The compression system of the isothermal piston is shown in Figure 6b. Parameters of the experimental setup are shown in Table 2.

## 4. Results and Discussion

The effectiveness of the isothermal piston should be tested by experiments. Therefore, two experiments were conducted: an isothermal experiment (with isothermal piston) and adiabatic experiment (with conventional piston). The effectiveness of the isothermal piston can be verified intuitively through the *p–V* diagram. The air pressure will be lower if more heat is transferred. However, the pressure will also be lower due to the mass loss of compressed air. To verify the heat transfer effect of the isothermal piston, the pressure drop caused by the mass loss of air should be compensated, which is caused by dissolution and leakage in the compression process.

### 4.1. Compensation for Dissolution

**Figure 7:**
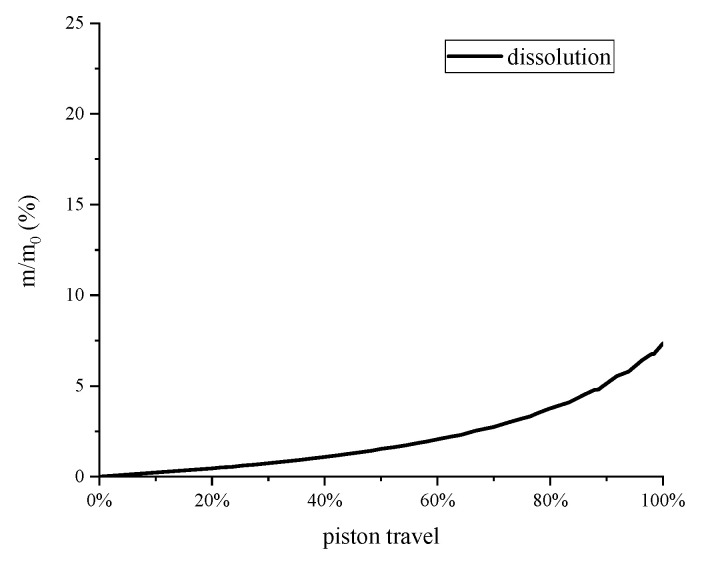
Dissolution rate of the isothermal experiment.

The air will be dissolved into the water as the compression proceeds. Air mainly consists of O_2_ (22%) and N_2_ (78%), and Henry’s law constant is 1.3 × 10^−3^ and 6.1 × 10^−4^, respectively. Based on Equation (7), converting molarity to mass and water volume, the amount of dissolved air can be obtained as follows:

(9) $$dm_{dis}=dp_{iso\_\exp}\cdot H^{cp}\cdot V_{water}$$

where $m_{dis}$ is the mass of dissolved air and $V_{water}$ is the volume of water in the compression chamber. The dissolution rate of the isothermal experiment is shown in Figure 7.

### 4.2. Compensation for Leakage

**Figure 8:**
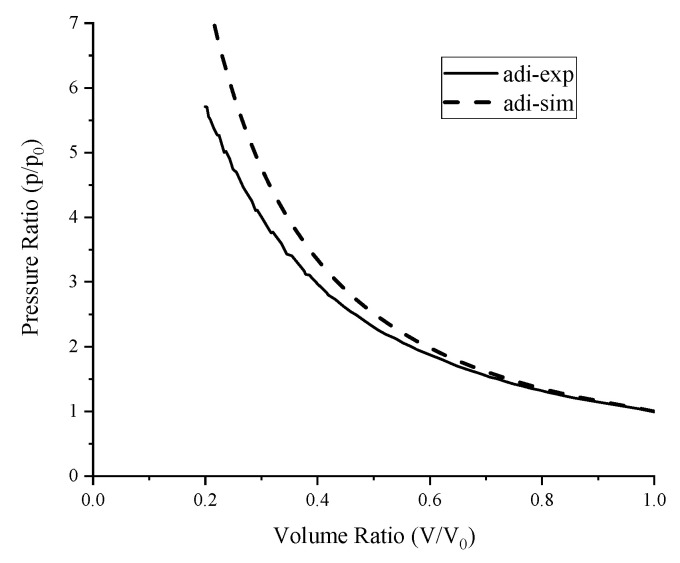
Pressure drop by leakage in the adiabatic experiment.

As the compression speed is so fast in one cycle, the heat transferred to the ambient can be ignored, so the adiabatic experiment can be treated as adiabatic compression, and *δQ* is 0. Under this condition, the pressure drop between the experiment and simulation was caused by leakage, as shown in Figure 8. The amount of air leakage can be estimated by Equation (10):

(10) $$\Delta m_{lea}=\frac{\left(p^{\prime }{}_{adi\_sim}-p_{adi\_\exp}\right)\cdot V}{R\cdot T_{adi}}$$

where $p_{adi\_sim}^{\prime }$ is the adiabatic pressure compensated by leakage. According to the state equation, energy equation and Henry’s law, it is obtained as follows:

(11) $$\left\{ \begin{array}{l} dU=dH_{dis}+\delta W\\ d\left(p_{adi\_sim}^{\prime }\cdot V\right)=d\left(m\cdot R\cdot T\right)\\ dm=-H^{cp}\cdot V_{water}\cdot dp_{adi\_sim}^{\prime } \end{array}\right.$$

**Figure 9:**
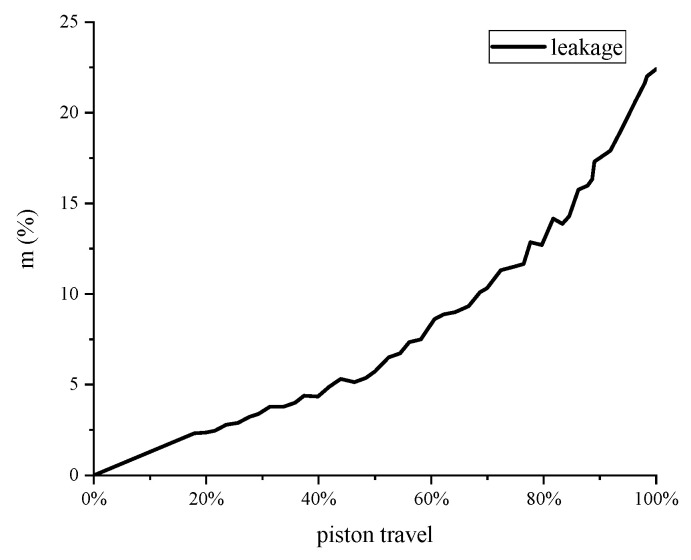
Leakage rate of the isothermal experiment.

The leakage rate of the two experiments were assumed to be the same by piston travel. The leakage rate of the isothermal experiment is shown in Figure 9.

### 4.3. Friction Analysis

**Figure 10:**
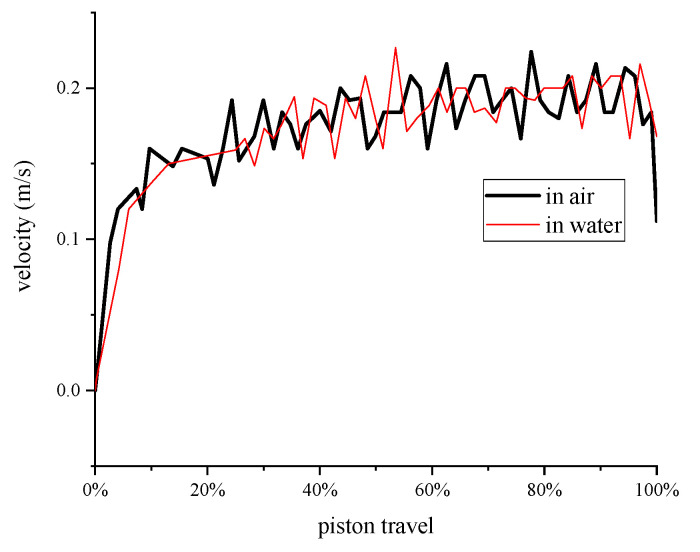
Piston velocity.

The air flow was set the same for the two contrast experiments in order to make the speed of the piston the same. The piston speed was 0.2 m/s, as shown in Figure 10. The piston speed of the two experiments agreed well.

**Figure 11:**
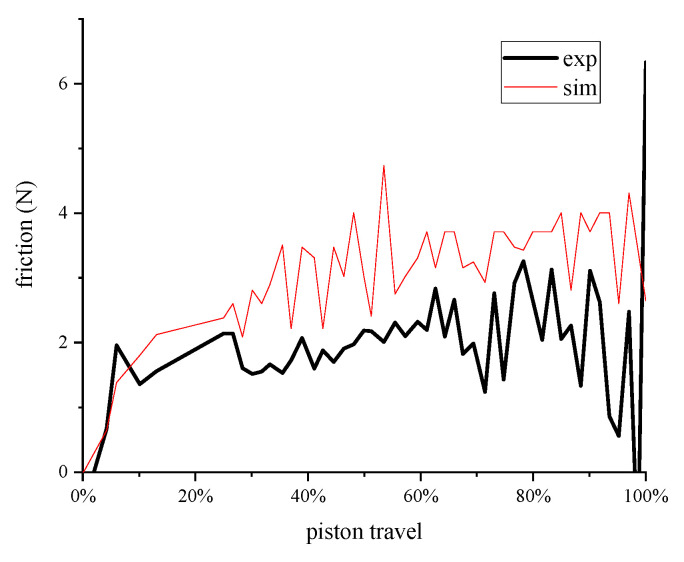
Resistance between porous media and water.

The resistance measured by the experiment and the results calculated by the model are shown in Figure 11. There were some fluctuations in the measured resistance value, but the overall resistance value was around 2.5 N, and the measured values were all smaller than the calculated value of the model. The reason may be that the model is an empirical model, which is different from the actual working condition, thus it can be used as a reference.

### 4.4. Validation of Effectiveness

**Figure 12:**
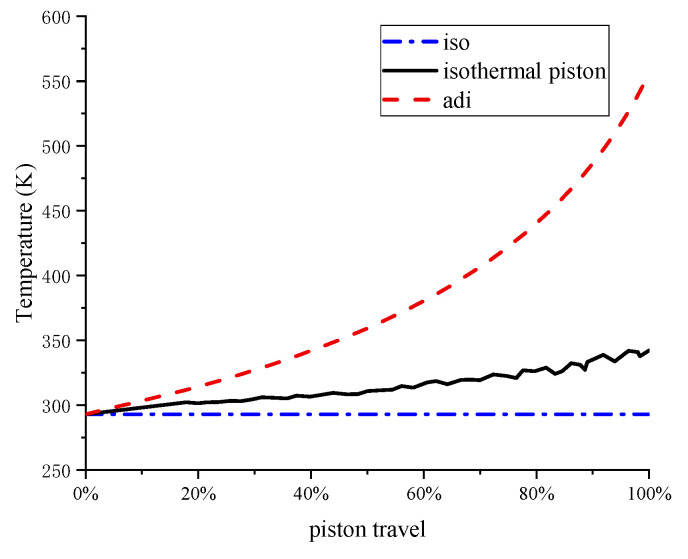
Temperature of the air as a function of piston travel.

The mass of the remaining air in the chamber can be acquired as:

$$m_{remain}=m_{0}-\Delta m_{dis}-\Delta m_{lea}$$

where *m*_0_ is the initial mass of air. The measurement of transient temperature is challenging while compressing, as the response time of temperature sensors cannot be in the order of milliseconds. From the pressure, volume, and mass of the air, the temperature in the isothermal experiment can be calculated based on the ideal gas law as follows, which is shown in Figure 12.

(12) $$T_{iso\_\exp}=\frac{p_{iso\_\exp}\cdot V_{air}}{m_{remain}\cdot R}$$

The pressure drop caused by dissolution and leakage is $\Delta p_{dis}$ and $\Delta p_{lea}$, respectively, which are expressed as:

(13) $$\Delta p_{dis}=\frac{\Delta m_{dis}\cdot R\cdot T_{iso\_\exp}}{V_{air}}$$

(14) $$\Delta p_{lea}=\frac{\Delta m_{lea}\cdot R\cdot T_{iso\_\exp}}{V_{air}}$$

The air pressure of the isothermal experiment compensated by dissolution and leakage $p_{iso\_com}$ is:

(15) $$p_{iso\_com}=p_{iso\_\exp}+\Delta p_{dis}+\Delta p_{lea}$$

**Figure 13:**
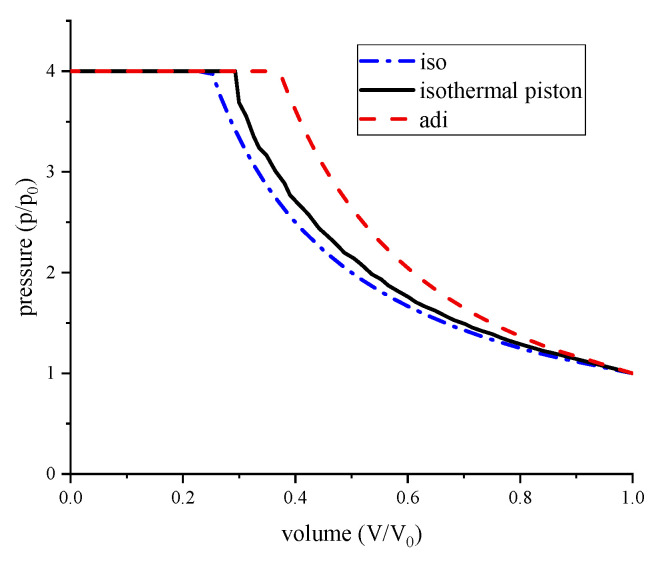
Air pressure in the chamber during the compression process.

The air pressure is shown in Figure 13, which increased from atmospheric pressure to the compression ratio, at which point the air is pumped at a constant pressure out of the chamber. Note that due to the good heat transfer of the isothermal piston, the air pressure is lower, and the pressure in the isothermal piston chamber rises far slower than the conventional piston chamber.

The work required to compress the air can be computed by numerically integrating the product of the change of volume times the difference of the air pressure minus the atmospheric pressure.

**Figure 14:**
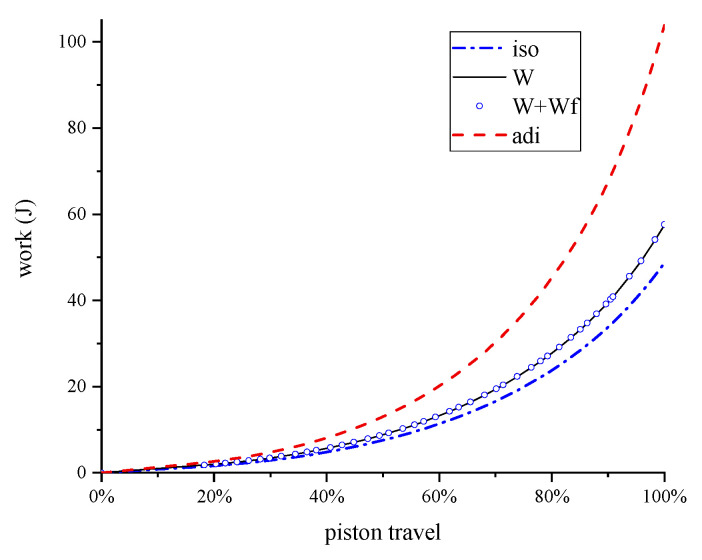
Compression work done on the air as a function of piston travel. This plot includes the work to overcome the frictional forces.

The compression work, as seen in Figure 14, was lower than the adiabatic compression due to the enhancement of heat transfer. Considering the frictional work, the total work done on the system is also presented. The effect of frictional forces on total work was relatively small.

### 4.5. Discussion

The isothermal piston illustrates a significant improvement in gas compression efficiency by using an isothermal piston in place of a conventional one. From this study, the isothermal piston chamber required 45% less work than the conventional piston chamber. The improvement was the result of removing more heat during the compression process, thus lowering the work required to compress the gas. Compressed air is stored in a pressure vessel before use, and will cool to near ambient temperature during storage. Therefore, any heat energy added to the gas during compression is lost. From this point, conducting a near-isothermal compression with an isothermal piston requires less work to compress the gas to the same final pressure than a near-adiabatic compression.

Near-isothermal compression is accomplished in the isothermal piston chamber by maximizing the heat transfer area via the structure of a porous medium. The porous medium has a large specific surface area and porosity, which provides a larger heat transfer area. As copper is a good conductor of heat, the compression heat can be rapidly conducted away. Unfortunately, resistance of frictional forces can be increased as the porous medium proceeds in the liquid.

The work to overcome frictional forces is negligible on such conditions of the experiments, but the influence cannot be neglected for the investigation of an isothermal piston. When the operation speed is relatively high, the resistance will increase significantly.

It does need to be clearly stated that this paper made assumptions to simplify the analysis and illustrate the isothermal piston concept. The temperature will be affected by dissolution and leakage. However, the effect is slight because the rate of mass loss is negligible compared to the remaining mass of air. When compensating, the effect of mass loss of air on temperature is ignored. The air temperature in an adiabatic experiment is assumed as the same as the simulation, and is regarded to have no effect on the dissolution and leakage when calculating the air temperature.

Near-isothermal compression with good heat transfer makes the isothermal piston concept advantageous in many applications. While this paper focused on a single application of an air compressor, this concept can also be used for condensing. The working medium can be the cooling liquid for the isothermal piston.

## 5. Conclusions

In the energy consumption distribution of a fluid power system, energy loss of pneumatic systems account for a fairly large portion. The energy loss of pneumatic systems is mainly in the pipelines and compressors. The energy loss in pipelines is mainly caused by leakage and pressure loss, while in compressors, the energy loss is mainly from the heat loss in the compression of a gas. Based on this, this paper proposed an isothermal compression method using an isothermal piston. Considering the influence of gas leakage and dissolution, the effectiveness was validated.

Isothermal piston compression is an exciting concept that can significantly lower the energy consumption of gas compression. This concept was demonstrated to lower the total energy consumption by 45% at the pressure ratio of 4:1. The air temperature was lowered from 517 K to 342 K. The effect of frictional forces was negligible (0.21% of the compression work) at the speed of 100 rpm.

The isothermal piston concept relies on maximizing the heat transfer area via the porous medium, allowing the rapid heat transfer from a gas to a liquid.

While the total energy consumption can be lowered with an isothermal piston, it operates at a relatively low speed compared with the actual conditions of a compressor. How frictional forces will affect the total energy consumption at high speed should be investigated. Further future work also includes the optimum design of an isothermal piston and an exploration of its applications.

## Figures and Tables

**Table 1:** Copper foam parameters.

Parameter Name	Symbol	Parameter
Porosity of the foam copper	*ε*/%	0.92
Hole density	*ppi*	40
Specific surface area of the foam copper	*S_v_*/m^−1^	2980
Volume of the foam copper	*V_por_*/cm^3^	30

**Table 2:** Parameters of the experimental setup.

Equipment	Company/Serial No.	Parameter
Compression chamber	-	*Φ*100 mm, L200 mm
Drive cylinder	SMC/CDQ2B100-100D	*Φ*100 mm, L120 mm
Pressure regulator	SMC/AF30-03	0.1–1.0 MPa
Solenoid valve	SMC/SY5140-5L-02 <0.15–0.7 MPa>	Frequency < 20 Hz
Displacement sensor	SiFang	Resolution: 40 μm/pulse Range: 0–1 m
Pressure sensor	KELLER/PR-25	Range: −1–10 bar Accuracy: ±0.2% FS Frequency < 5 kHz

## Abbreviations

*c*	molarity (mol/L)	Greek symbols	
*C_c_*	Inertia coefficient of porous medium	*ε*	Porosity (%)
*d*	Hydraulic radius of a porous medium (m)	*ρ*	Density of fluids (kg/m^3^)
*H*	Enthalpy (J)	*μ*	Dynamic viscosity (Pa∙s)
*H^cp^*	Henry’s Law constant (mol/L∙atm)	Subscripts & Superscripts	
*K*	Permeability of porous medium (%)	0	Initial condition
*m*	Mass (kg)	*adi*	Adiabatic condition
*p*	Pressure (Pa)	*dis*	Dissolution
*R*	Gas constant (J/kg·K^−1^)	exp	Experiment
*T*	Temperature (K)	f	friction
*u*	Relative velocity between a fluid and a porous medium (m/s)	*iso*	Isothermal condition
*U*	Internal energy (J)	*lea*	Leakage
*V*	Volume (m^3^)	*sim*	Simulation
*W*	Work (J)		

## References

[1] Global CO_2_ Emissions in 2019. https://www.iea.org/articles/global-co2-emissions-in-2019.

[2] Kabeel A., Abdelgaied M., Al Ali M. (2018). Energy saving potential of a solar assisted desiccant air conditioning system for different types of storage. Environ. Prog. Sustain. Energy.

[3] Liu Y., Zhou Y., Wu W. (2015). Assessing the impact of population, income and technology on energy consumption and industrial pollutant emissions in China. Appl. Energy.

[4] Sun Y., Li Y., Cai B.-F., Li Q. (2020). Comparing the explicit and implicit attitudes of energy stakeholders and the public towards carbon capture and storage. J. Clean. Prod..

[5] Kopetz H. (2013). Build a biomass energy market. Nature.

[6] Moog O. (1993). Quantification of daily peak hydropower effects on aquatic fauna and management to minimize environmental impacts. Regul. Rivers Res. Manag..

[7] IAEA (2000). Safety Standards Series No. NS-R-I. Safety of Nuclear Power Plants: Design: Safety Requirements.

[8] Mwasilu F., Justo J.J., Kim E.-K., Do T.D., Jung J.-W. (2014). Electric vehicles and smart grid interaction: A review on vehicle to grid and renewable energy sources integration. Renew. Sustain. Energy Rev..

[9] Scharlemann J.P., Laurance W.F. (2008). Environmental science: How green are biofuels?. Science.

[10] Grill G., Lehner B., Thieme M., Geenen B., Tickner D., Antonelli F., Babu S., Borrelli P., Cheng L., Crochetiere H. (2019). Mapping the world’s free-flowing rivers. Nature.

[11] Piera M. (2010). Sustainability issues in the development of Nuclear Fission energy. Energy Convers. Manag..

[12] Silvennoinen P. (2013). Nuclear Fuel Cycle Optimization: Methods and Modelling Techniques.

[13] Destek M.A., Aslan A. (2017). Renewable and non-renewable energy consumption and economic growth in emerging economies: Evidence from bootstrap panel causality. Renew. Energy.

[14] Gibbins J., Chalmers H. (2008). Carbon capture and storage. Energy Policy.

[15] Yao X., Zhong P., Zhang X., Zhu L. (2018). Business model design for the carbon capture utilization and storage (CCUS) project in China. Energy Policy.

[16] Peters G., Andrew R., Canadell J., Friedlingstein P., Jackson R., Korsbakken J., Le Quéré C., Peregon A. (2020). Carbon dioxide emissions continue to grow amidst slowly emerging climate policies. Nat. Clim. Chang..

[17] Lin C., Nakamura S. (2019). Approaches to solving China’s marine plastic pollution and CO_2_ emission problems. Econ. Syst. Res..

[18] Shi Y., Cai M., Xu W., Wang Y. (2019). Methods to evaluate and measure power of pneumatic system and their applications. Chin. J. Mech. Eng..

[19] Maolin C., Kagawa T. (2007). Energy consumption assessment and energy loss analysis in pneumatic system. Chin. J. Mech. Eng..

[20] Coney M.W., Stephenson P., Malmgren A., Linnemann C., Morgan R., Richards R., Huxley R., Abdallah H. Development of a reciprocating compressor using water injection to achieve quasi-isothermal compression. Proceedings of the 2002 International Compressor Engineering Conference.

[21] Saadat M., Li P.Y. Combined optimal design and control of a near isothermal liquid piston air compressor/expander for a compressed air energy storage (CAES) system for wind turbines. Proceedings of the ASME 2015 Dynamic Systems and Control Conference.

[22] McBride T., Bell A., Kepshire D. (2013). ICAES Innovation: Foam-Based Heat Exchange. http://www.sustainx.com.

[23] Guanwei J., Weiqing X., Maolin C., Yan S. (2018). Micron-sized water spray-cooled quasi-isothermal compression for compressed air energy storage. Exp. Therm. Fluid Sci..

[24] Wieberdink J., Li P.Y., Simon T.W., Van de Ven J.D. (2018). Effects of porous media insert on the efficiency and power density of a high pressure (210 bar) liquid piston air compressor/expander—An experimental study. Appl. Energy.

[25] Wieberdink J.H. (2014). Increasing Efficiency and Power Density of a Liquid Piston Air Compressor/Expander with Porous Media Heat Transfer Elements. Master’s Thesis.

[26] Yan B., Wieberdink J., Shirazi F., Li P.Y., Simon T.W., Van de Ven J.D. (2015). Experimental study of heat transfer enhancement in a liquid piston compressor/expander using porous media inserts. Appl. Energy.

[27] Van de Ven J.D., Li P.Y. (2009). Liquid piston gas compression. Appl. Energy.

[28] Ashby M.F., Lu T. (2003). Metal foams: A survey. Sci. China Ser. B Chem..

[29] Dukhan N., Minjeur C.A. (2011). A two-permeability approach for assessing flow properties in metal foam. J. Porous Mater..

[30] Sivanesapillai R., Steeb H., Hartmaier A. (2014). Transition of effective hydraulic properties from low to high Reynolds number flow in porous media. Geophys. Res. Lett..

[31] Schweitzer P., Szebehely V.G. (1950). Gas evolution in liquids and cavitation. J. Appl. Phys..

[32] Ren T., Xu W., Cai M., Wang X., Li M. (2019). Experiments on Air Compression with an Isothermal Piston for Energy Storage. Energies.

